# Supplementary material for: Is Continuous ECG Recording on Heart Rate Monitors the Most Expected Function by Endurance Athletes, Coaches, and Doctors?
Source: Diagnostics (Basel). 2020 Oct 23;10(11):867. doi: 10.3390/diagnostics10110867 (PMC7690815; doi:10.3390/diagnostics10110867)
Supplement: Supplementary file 1 [file diagnostics-10-00867-s001.pdf]

Supplementary Materials:

Table S1.1. Cumulative results of the study groups (athlete, coaches, doctors) including function, sport discipline, gender, age, experience with HRMS, and answers to 11 questions in two situations of healthy athletes or those suspected of arrhythmia.

Respondent	Sport discipline	Gender [Male/ Female]	Age [years]	Experience with OHRMS [years]	Experience with SHRMS [years]	H/A	F1	F2	F3	F4	F5	F6	F7	F8	F9	F10	F11
Coach 1	N/A	M	45	0.5	6.5	H	1	3	4	5	10	7	8	2	9	11	6
Coach 2	N/A	M	51	3.5	6	H	1	2	3	5	11	7	9	4	8	10	6
Coach 3	N/A	M	48	2	5	H	1	2	3	7	10	5	8	4	9	11	6
Coach 4	N/A	M	54	3.5	5.5	H	1	2	3	5	10	8	7	4	9	11	6
Coach 5	N/A	M	26	1	4	H	1	2	3	5	10	7	8	4	6	11	9
Coach 6	N/A	M	51	2.5	8	H	2	3	1	5	10	7	8	4	9	11	6
Coach 7	N/A	M	46	3	8.5	H	1	2	4	5	10	7	8	3	9	11	6
Coach 8	N/A	M	36	3	9.5	H	1	2	3	6	9	7	10	4	8	11	5
Coach 9	N/A	M	60	2	10	H	1	2	3	5	11	8	7	4	9	10	6
Coach 10	N/A	M	34	4	3	H	1	2	3	5	11	6	8	4	10	9	7
Coach 1	N/A	M	45	0.5	6.5	A	5	1	2	9	11	4	3	6	10	8	7
Coach 2	N/A	M	51	3.5	6	A	4	1	2	6	9	5	3	11	10	8	7
Coach 3	N/A	M	48	2	5	A	5	3	2	7	11	4	1	6	9	8	10
Coach 4	N/A	M	54	4	5.5	A	4	1	5	9	7	2	3	6	10	8	11
Coach 5	N/A	M	26	1	4	A	5	2	1	9	8	4	3	6	10	11	7
Coach 6	N/A	M	51	3	8	A	5	1	2	9	11	4	3	10	6	8	7
Coach 7	N/A	M	46	3	8.5	A	3	1	2	9	11	4	5	6	7	8	10
Coach 8	N/A	M	36	3	9.5	A	5	4	2	8	11	1	3	6	10	9	7
Coach 9	N/A	M	60	2	10	A	5	1	2	9	11	4	3	7	10	8	6

Coach 10	N/A	M	34	4	3	A	5	1	4	11	10	2	3	6	9	8	7
Doctor 1	N/A	M	43	0.5	4	H	8	9	6	7	10	2	1	5	11	3	4
Doctor 2	N/A	M	51	4	5	H	7	8	6	9	10	2	1	5	11	4	3
Doctor 3	N/A	M	39	1.5	5.5	H	8	9	7	6	10	2	1	5	11	4	3
Doctor 4	N/A	M	59	2	7	H	9	8	6	7	10	2	1	5	11	4	3
Doctor 5	N/A	M	49	1.5	6.5	H	7	9	6	8	10	2	1	5	11	4	3
Doctor 6	N/A	M	60	4	9	H	8	9	6	7	10	2	1	5	11	4	3
Doctor 7	N/A	M	55	3.5	5.5	H	8	9	4	7	11	3	1	6	10	5	2
Doctor 8	N/A	M	33	1	5	H	8	9	6	7	10	1	2	5	11	4	3
Doctor 9	N/A	M	57	3	4.5	H	8	10	5	7	9	2	1	4	11	6	3
Doctor 10	N/A	M	53	3	10	H	8	9	6	7	10	2	1	5	11	3	4
Doctor 1	N/A	M	43	0.5	4	A	7	6	5	8	11	2	1	9	10	4	3
Doctor 2	N/A	M	51	4	5	A	7	6	5	8	10	2	1	9	11	4	3
Doctor 3	N/A	M	39	1.5	5.5	A	6	7	5	8	11	2	1	9	10	4	3
Doctor 4	N/A	M	59	2	7	A	9	6	5	7	11	2	1	8	10	4	3
Doctor 5	N/A	M	49	1.5	6.5	A	7	5	6	8	11	2	1	9	10	4	3
Doctor 6	N/A	M	60	4	9	A	7	6	5	8	11	2	1	9	10	4	3
Doctor 7	N/A	M	55	3.5	5.5	A	8	6	5	9	11	2	1	7	10	4	3
Doctor 8	N/A	M	33	1	5	A	7	6	5	8	11	2	1	9	10	3	4
Doctor 9	N/A	M	57	3	4.5	A	7	6	5	8	11	2	1	9	10	4	3
Doctor 10	N/A	M	53	3	10	A	7	5	6	8	11	2	1	9	10	4	3
Athlete	Runner 1	M	35	0.5	5	H	1	2	3	5	7	8	9	4	10	11	6
Athlete	Runner 2	M	31	3	2	H	1	2	3	5	7	8	9	4	10	11	6
Athlete	Runner 3	F	25	2	2.5	H	1	2	3	5	7	8	10	4	9	11	6
Athlete	Runner 4	M	35	0.75	6	H	1	3	2	5	7	8	9	4	10	11	6
Athlete	Runner 5	F	37	4	5	H	1	2	3	5	7	8	9	4	10	11	6

Athlete	Runner 6	F	39	1	5.75	H	1	2	3	5	7	9	11	4	8	10	6
Athlete	Runner 7	M	36	2	4	H	1	2	4	5	7	8	9	3	10	11	6
Athlete	Runner 8	M	41	1	9.5	H	1	2	3	5	7	8	10	4	9	11	6
Athlete	Runner 9	M	43	3	8	H	1	2	3	5	7	8	9	4	10	11	6
Athlete	Runner 10	F	28	3	2.5	H	1	2	3	5	7	8	9	4	10	11	6
Athlete	Runner 11	M	35	2	9	H	1	2	3	4	7	8	9	5	10	11	6
Athlete	Runner 12	M	29	1	3.5	H	1	2	3	5	7	8	9	4	10	11	6
Athlete	Runner 13	M	36	1.25	3.25	H	1	2	3	5	7	9	11	4	8	10	6
Athlete	Runner 14	M	37	1	10	H	1	3	2	5	7	8	9	4	10	11	6
Athlete	Runner 15	F	42	1.25	5.25	H	1	2	3	6	7	8	10	4	9	11	5
Athlete	Runner 16	M	36	3	8	H	1	2	3	5	7	9	8	4	11	10	6
Athlete	Runner 17	M	29	1	4.5	H	1	2	5	3	7	8	9	4	10	11	6
Athlete	Runner 18	M	29	2	5.5	H	1	2	3	5	7	8	9	4	10	11	6
Athlete	Runner 19	F	39	1	5	H	1	2	3	5	7	9	11	4	8	10	6
Athlete	Runner 20	M	27	3	2.75	H	1	2	3	5	7	8	9	4	10	11	6
Athlete	Runner 21	M	34	2	3	H	1	2	3	5	6	8	9	4	10	11	7
Athlete	Runner 22	F	39	2	8	H	1	2	3	4	7	8	9	5	10	11	6
Athlete	Runner 23	M	41	0.75	7.5	H	1	2	4	5	7	9	8	3	11	10	6
Athlete	Runner 24	M	21	0.25	2	H	1	2	3	5	7	8	9	4	10	11	6
Athlete	Runner 25	M	43	1	5.25	H	1	2	4	5	7	8	9	3	10	11	6
Athlete	Runner 26	F	44	3	6.5	H	1	2	3	5	7	8	9	4	10	11	6
Athlete	Runner 27	M	28	1	3.25	H	1	2	3	6	7	8	10	4	9	11	5
Athlete	Runner 28	M	31	0.5	3	H	1	2	3	5	7	8	9	4	10	11	6
Athlete	Runner 29	M	32	1.25	4	H	1	2	3	5	7	10	8	4	11	9	6
Athlete	Runner 30	M	35	1	7	H	2	1	3	5	7	8	9	4	10	11	6
Athlete	Runner 31	F	38	0.25	6.5	H	1	2	3	5	7	8	9	4	10	11	6

Athlete	Runner 32	M	36	1	4.25	H	1	2	3	5	7	6	9	4	10	11	8
Athlete	Runner 33	M	38	1.5	9	H	1	2	5	3	7	8	9	4	10	11	6
Athlete	Runner 34	M	28	1	3.75	H	1	2	3	5	7	8	9	4	10	11	6
Athlete	Runner 35	F	31	1	6.5	H	1	2	3	5	6	9	8	4	11	10	7
Athlete	Runner 36	M	27	3.5	2.5	H	1	2	3	5	7	8	9	4	10	11	6
Athlete	Runner 37	F	34	1	7.5	H	1	2	3	5	7	8	10	4	9	11	6
Athlete	Runner 38	M	34	1	8.5	H	1	2	5	3	7	8	9	4	10	11	6
Athlete	Runner 39	M	40	2	4.25	H	1	2	3	5	7	10	8	4	11	9	6
Athlete	Runner 40	F	42	2	6.25	H	1	2	3	5	7	8	9	4	10	11	6
Athlete	Runner 41	M	57	1	10	H	1	2	3	5	6	8	9	4	10	11	7
Athlete	Runner 42	F	26	3	2	H	1	2	3	5	7	8	9	4	11	10	6
Athlete	Runner 43	F	33	2	5.5	H	1	2	3	5	7	8	9	4	10	11	6
Athlete	Runner 44	M	30	3	2.5	H	2	1	3	5	7	8	9	4	10	11	6
Athlete	Runner 45	F	33	1	3.5	H	1	2	3	7	5	8	9	4	10	11	6
Athlete	Runner 46	F	36	2	5	H	1	2	3	5	7	9	8	4	11	10	6
Athlete	Runner 47	M	44	1	5.25	H	1	2	3	5	7	8	9	4	10	11	6
Athlete	Runner 48	M	33	2.5	6.5	H	1	4	2	5	7	8	9	3	11	10	6
Athlete	Runner 49	M	38	2	9.25	H	1	2	3	5	7	8	9	4	10	11	6
Athlete	Runner 50	F	36	1	7.5	H	1	2	3	5	7	8	10	4	9	11	6
Athlete	Runner 51	M	36	3	7	H	1	2	3	5	7	6	9	4	10	11	8
Athlete	Runner 52	M	50	0.75	10	H	1	2	3	5	7	10	8	4	11	9	6
Athlete	Runner 53	F	45	0.5	6	H	1	2	3	4	7	8	9	5	10	11	6
Athlete	Runner 54	M	37	2	5	H	1	2	3	5	7	8	10	4	9	11	6
Athlete	Runner 55	M	37	2	6	H	1	2	3	5	6	8	9	4	10	11	7
Athlete	Runner 56	M	38	0.5	4.25	H	2	1	3	5	7	8	9	4	10	11	6
Athlete	Runner 57	M	40	0.25	8	H	1	2	3	5	7	8	9	4	10	11	6

Athlete	Runner 58	M	35	0.25	4	H	1	2	3	5	7	9	8	4	11	10	6
Athlete	Runner 59	F	36	2.5	6	H	1	2	3	5	7	8	9	4	10	11	6
Athlete	Runner 60	F	25	1.75	3.25	H	1	2	3	7	6	8	9	4	10	11	5
Athlete	Runner 61	M	31	0.75	5.5	H	1	2	3	5	7	8	10	4	9	11	6
Athlete	Runner 62	M	25	1.5	5.25	H	1	2	3	5	7	8	9	4	10	11	6
Athlete	Runner 63	F	31	0.25	3.5	H	1	2	3	5	7	8	10	4	9	11	6
Athlete	Runner 64	M	35	2	8	H	1	2	3	5	7	8	9	4	10	11	6
Athlete	Runner 65	M	28	2	2.25	H	1	2	3	5	7	8	10	4	9	11	6
Athlete	Runner 66	M	29	3	2.5	H	1	2	3	5	7	10	8	4	11	9	6
Athlete	Runner 67	M	38	3	6	H	1	2	3	5	7	8	9	4	10	11	6
Athlete	Runner 68	M	39	1	9	H	1	2	3	5	7	8	9	4	10	11	6
Athlete	Runner 69	F	30	1	4.25	H	1	2	3	5	6	7	9	4	10	11	8
Athlete	Runner 70	M	51	1	9	H	1	2	3	5	7	8	10	4	9	11	6
Athlete	Runner 71	F	22	0.5	3	H	1	3	2	5	7	8	9	4	10	11	6
Athlete	Runner 72	M	34	2	2	H	1	2	3	5	7	9	8	4	11	10	6
Athlete	Runner 73	F	34	1	9.5	H	1	2	3	5	7	8	9	4	10	11	6
Athlete	Runner 74	F	26	2.5	4.5	H	1	2	3	5	7	8	9	4	10	11	6
Athlete	Runner 75	M	43	2	9.5	H	1	2	3	5	7	8	9	4	10	11	6
Athlete	Runner 76	F	24	1	2.25	H	1	2	3	5	6	8	9	4	10	11	7
Athlete	Cyclist 1	M	24	1.5	2.25	H	1	2	3	5	7	8	9	4	10	11	6
Athlete	Cyclist 2	M	37	3	7	H	1	2	3	5	7	10	8	4	11	9	6
Athlete	Cyclist 3	M	27	2	5	H	1	2	3	5	7	8	9	4	10	11	6
Athlete	Cyclist 4	M	48	0.5	8.5	H	1	2	3	5	7	8	10	4	9	11	6
Athlete	Cyclist 5	M	26	3	3	H	1	2	3	5	7	8	9	4	10	11	6
Athlete	Cyclist 6	M	37	1	9	H	1	3	2	6	7	8	9	4	10	11	6
Athlete	Cyclist 7	M	39	3	10	H	1	2	3	5	6	7	9	4	10	11	8

Athlete	Cyclist 8	F	55	1	6.25	H	1	2	3	5	7	8	9	4	10	11	6
Athlete	Cyclist 9	M	38	0.75	7	H	1	2	3	5	7	8	9	6	10	11	6
Athlete	Cyclist 10	F	38	1	7.25	H	1	2	3	5	7	11	10	4	9	8	6
Athlete	Cyclist 11	M	40	2	5.25	H	1	2	3	5	7	8	9	4	10	11	6
Athlete	Cyclist 12	M	30	2	3.25	H	1	2	3	5	7	8	10	4	9	11	6
Athlete	Cyclist 13	M	30	2	3.25	H	1	2	3	7	6	8	9	4	10	11	5
Athlete	Cyclist 14	M	33	2	4.5	H	1	2	4	5	7	9	8	4	11	10	6
Athlete	Triathlete 1	M	40	4	7	H	1	2	3	5	7	8	9	4	10	11	6
Athlete	Triathlete 2	F	24	0.75	2.25	H	1	2	3	5	7	8	9	4	10	11	6
Athlete	Triathlete 3	M	27	1	4	H	1	2	3	5	7	8	10	4	9	11	6
Athlete	Triathlete 4	M	32	1.75	2.5	H	2	1	3	6	7	8	9	4	10	11	5
Athlete	Triathlete 5	M	39	1.75	6.75	H	1	2	3	5	7	8	10	4	9	11	6
Athlete	Triathlete 6	M	32	1.25	4.25	H	1	2	5	3	7	8	9	4	10	11	6
Athlete	Triathlete 7	M	39	2	9	H	1	2	3	5	7	11	10	4	9	8	6
Athlete	Triathlete 8	M	40	1	10	H	2	1	3	7	6	8	9	4	10	11	5
Athlete	Triathlete 9	M	41	0.5	8.5	H	1	2	3	5	7	9	8	4	11	10	6
Athlete	Triathlete 10	M	38	1	6.25	H	1	2	4	5	7	8	9	3	10	11	6
Athlete	Runner 1	M	35	0.5	5	A	1	2	3	8	10	5	4	9	11	7	6
Athlete	Runner 2	M	31	3	2	A	1	2	3	7	10	5	4	9	11	8	6
Athlete	Runner 3	F	25	2	2.5	A	1	2	3	7	10	5	4	9	11	8	6
Athlete	Runner 4	M	35	0.75	6	A	2	1	3	7	10	5	4	8	11	9	6
Athlete	Runner 5	F	37	4	5	A	1	2	5	7	11	3	4	9	8	10	6
Athlete	Runner 6	F	39	1	5.75	A	1	2	3	7	10	5	4	9	11	8	6
Athlete	Runner 7	M	36	2	4	A	2	1	3	7	9	5	4	10	8	11	6
Athlete	Runner 8	M	41	1	9.5	A	1	2	3	7	10	4	5	9	11	8	6

Athlete	Runner 9	M	43	3	8	A	1	2	5	7	10	3	4	9	11	8	6
Athlete	Runner 10	F	28	3	2.5	A	3	1	2	7	11	5	4	9	8	10	6
Athlete	Runner 11	M	35	2	9	A	1	2	3	7	10	5	6	9	11	8	4
Athlete	Runner 12	M	29	1	3.5	A	1	2	3	7	10	5	4	8	11	9	6
Athlete	Runner 13	M	36	1.25	3.25	A	1	2	7	5	10	3	4	9	11	8	6
Athlete	Runner 14	M	37	1	10	A	2	1	3	7	10	5	4	9	11	8	6
Athlete	Runner 15	F	42	1.25	5.25	A	1	2	3	7	11	5	4	9	8	10	6
Athlete	Runner 16	M	36	3	8	A	3	1	2	7	10	5	4	9	11	8	6
Athlete	Runner 17	M	29	1	4.5	A	3	1	2	4	10	5	6	9	11	8	7
Athlete	Runner 18	M	29	2	5.5	A	1	2	3	7	10	5	4	9	11	8	6
Athlete	Runner 19	F	39	1	5	A	2	1	3	7	9	5	4	10	8	11	6
Athlete	Runner 20	M	27	3	2.75	A	1	2	5	7	10	3	4	9	11	8	6
Athlete	Runner 21	M	34	2	3	A	1	2	3	7	10	5	4	9	11	8	6
Athlete	Runner 22	F	39	2	8	A	1	2	3	7	11	5	4	9	8	10	6
Athlete	Runner 23	M	41	0.75	7.5	A	1	2	3	4	10	5	6	9	11	8	7
Athlete	Runner 24	M	21	0.25	2	A	1	2	7	3	10	5	4	9	11	8	6
Athlete	Runner 25	M	43	1	5.25	A	2	1	3	7	10	5	4	8	11	9	6
Athlete	Runner 26	F	44	3	6.5	A	1	2	3	7	10	5	4	9	11	8	6
Athlete	Runner 27	M	28	1	3.25	A	1	2	3	7	10	4	5	9	11	8	6
Athlete	Runner 28	M	31	0.5	3	A	2	1	3	7	10	5	4	9	11	8	6
Athlete	Runner 29	M	32	1.25	4	A	1	2	3	7	9	5	4	10	8	11	6
Athlete	Runner 30	M	35	1	7	A	1	2	3	7	10	5	4	9	11	8	6
Athlete	Runner 31	F	38	0.25	6.5	A	2	1	3	7	10	5	4	9	11	8	6
Athlete	Runner 32	M	36	1	4.25	A	1	2	3	6	10	5	4	9	11	8	7
Athlete	Runner 33	M	38	1.5	9	A	1	2	3	7	11	5	4	9	8	10	6
Athlete	Runner 34	M	28	1	3.75	A	1	2	3	7	10	5	4	9	11	8	6

Athlete	Runner 35	F	31	1	6.5	A	3	1	2	7	10	5	4	9	11	8	6
Athlete	Runner 36	M	27	3.5	2.5	A	1	2	3	7	9	5	4	10	8	11	6
Athlete	Runner 37	F	34	1	7.5	A	1	2	3	7	10	5	4	9	11	8	6
Athlete	Runner 38	M	34	1	8.5	A	2	1	3	7	10	5	4	9	11	8	6
Athlete	Runner 39	M	40	2	4.25	A	1	2	3	7	10	5	4	9	11	8	6
Athlete	Runner 40	F	42	2	6.25	A	1	2	7	3	10	5	4	9	11	8	6
Athlete	Runner 41	M	57	1	10	A	2	1	3	7	11	5	4	9	8	10	6
Athlete	Runner 42	F	26	3	2	A	1	2	3	7	10	5	4	9	11	8	6
Athlete	Runner 43	F	33	2	5.5	A	1	2	3	7	10	5	4	9	11	8	6
Athlete	Runner 44	M	30	3	2.5	A	1	2	3	7	10	5	4	9	11	8	6
Athlete	Runner 45	F	33	1	3.5	A	2	1	3	7	10	5	6	9	11	8	4
Athlete	Runner 46	F	36	2	5	A	1	2	3	7	9	5	4	10	8	11	6
Athlete	Runner 47	M	44	1	5.25	A	1	2	3	7	10	5	4	9	11	8	6
Athlete	Runner 48	M	33	2.5	6.5	A	1	2	3	8	10	5	4	9	11	8	6
Athlete	Runner 49	M	38	2	9.25	A	1	2	3	7	11	5	4	9	8	10	6
Athlete	Runner 50	F	36	1	7.5	A	1	2	3	7	10	5	4	9	11	8	6
Athlete	Runner 51	M	36	3	7	A	1	2	3	7	10	5	4	9	11	8	6
Athlete	Runner 52	M	50	0.75	10	A	2	1	3	7	10	5	4	9	11	8	6
Athlete	Runner 53	F	45	0.5	6	A	1	2	3	7	10	5	4	9	11	8	6
Athlete	Runner 54	M	37	2	5	A	1	2	3	5	10	6	4	9	11	8	7
Athlete	Runner 55	M	37	2	6	A	1	2	3	7	10	5	4	9	11	8	6
Athlete	Runner 56	M	38	0.5	4.25	A	1	2	5	7	10	3	4	9	11	8	6
Athlete	Runner 57	M	40	0.25	8	A	1	2	3	7	10	5	4	9	11	8	6
Athlete	Runner 58	M	35	0.25	4	A	3	1	2	7	11	5	4	9	8	10	6
Athlete	Runner 59	F	36	2.5	6	A	1	2	3	7	10	5	4	9	11	8	6
Athlete	Runner 60	F	25	1.75	3.25	A	1	2	7	3	10	5	4	9	11	8	6

Athlete	Runner 61	M	31	0.75	5.5	A	1	2	3	7	11	5	4	9	8	10	6
Athlete	Runner 62	M	25	1.5	5.25	A	1	2	3	7	10	5	4	9	11	8	6
Athlete	Runner 63	F	31	0.25	3.5	A	1	2	3	7	9	5	4	10	8	11	6
Athlete	Runner 64	M	35	2	8	A	2	1	3	7	10	5	6	9	11	8	4
Athlete	Runner 65	M	28	2	2.25	A	1	2	3	7	10	5	4	9	11	8	6
Athlete	Runner 66	M	29	3	2.5	A	1	2	3	7	10	5	4	9	11	8	6
Athlete	Runner 67	M	38	3	6	A	1	2	7	5	10	3	4	9	11	8	6
Athlete	Runner 68	M	39	1	9	A	1	2	3	7	10	5	4	9	11	8	6
Athlete	Runner 69	F	30	1	4.25	A	1	2	3	7	10	5	4	9	11	8	6
Athlete	Runner 70	M	51	1	9	A	1	2	3	7	10	5	4	9	11	8	6
Athlete	Runner 71	F	22	0.5	3	A	3	1	2	7	9	5	4	10	8	11	6
Athlete	Runner 72	M	34	2	2	A	1	2	3	7	10	5	4	9	11	8	6
Athlete	Runner 73	F	34	1	9.5	A	1	2	3	7	10	5	4	9	11	8	6
Athlete	Runner 74	F	26	2.5	4.5	A	1	2	3	7	11	5	4	9	8	10	6
Athlete	Runner 75	M	43	2	9.5	A	5	1	2	7	10	3	4	9	11	8	6
Athlete	Runner 76	F	24	1	2.25	A	1	2	3	7	10	5	4	9	11	8	6
Athlete	Cyclist 1	M	24	1.5	2.25	A	1	2	3	7	11	5	4	8	10	9	6
Athlete	Cyclist 2	M	37	3	7	A	2	1	8	3	10	5	4	9	11	7	6
Athlete	Cyclist 3	M	27	2	5	A	1	2	3	7	10	5	4	9	11	8	6
Athlete	Cyclist 4	M	48	0.5	8.5	A	1	2	3	7	10	5	4	9	11	8	6
Athlete	Cyclist 5	M	26	3	3	A	1	2	3	7	10	5	4	9	11	8	6
Athlete	Cyclist 6	M	37	1	9	A	3	1	2	7	9	5	4	10	8	11	6
Athlete	Cyclist 7	M	39	3	10	A	1	2	3	7	10	5	4	9	11	8	6
Athlete	Cyclist 8	F	55	1	6.25	A	1	2	7	3	10	5	4	9	11	8	6
Athlete	Cyclist 9	M	38	0.75	7	A	1	2	3	7	8	5	4	9	10	11	6
Athlete	Cyclist 10	F	38	1	7.25	A	1	2	3	7	10	5	4	9	11	8	6

Athlete	Cyclist 11	M	40	2	5.25	A	1	2	3	7	11	5	4	8	10	9	6
Athlete	Cyclist 12	M	30	2	3.25	A	1	2	3	4	10	5	6	9	11	8	7
Athlete	Cyclist 13	F	30	2	3.25	A	1	2	3	7	11	5	4	9	8	10	6
Athlete	Cyclist 14	M	33	2	4.5	A	3	1	2	7	10	5	4	9	11	8	6
Athlete	Triathlete 1	M	40	4	7	A	1	2	3	7	10	5	4	9	11	8	6
Athlete	Triathlete 2	F	24	0.75	2.25	A	1	2	3	7	11	5	4	8	10	9	6
Athlete	Triathlete 3	M	27	1	4	A	2	1	5	7	10	3	4	9	11	8	6
Athlete	Triathlete 4	M	32	1.75	2.5	A	1	2	3	7	10	5	4	9	11	8	6
Athlete	Triathlete 5	M	39	1.75	6.75	A	1	2	3	7	10	6	5	9	11	8	4
Athlete	Triathlete 6	M	32	1.25	4.25	A	1	2	3	7	11	5	4	9	8	10	6
Athlete	Triathlete 7	M	39	2	9	A	1	2	3	7	10	5	4	9	11	8	6
Athlete	Triathlete 8	M	40	1	10	A	2	1	3	7	11	5	4	9	8	10	6
Athlete	Triathlete 9	M	41	0.5	8.5	A	1	2	3	7	10	6	5	9	11	8	4
Athlete	Triathlete 10	M	38	1	6.25	A	1	2	3	7	10	5	4	9	11	8	6

Functions 1–11: F1, distance; F2, speed/pace; F3, current HR; F4, average training HR; F5, number of calories consumed during training (active kcal); F6, recording of the current ECG "on demand"; F7, continuous ECG recording; F8, the moment of reaching the anaerobic threshold (AT) (lactate threshold); F9, altitude (meters above sea level (MASL)); F10, heart rate variability (HRV); F11, 24-hour HR measurement. OHRM, optical Heart Rate Monitor; SHRM, strap Heart Rate Monitor; H/A, Healthy Athlete; A, Suspicion of arrhythmia; N/A, not applicable.

Table S1.2. Number of votes cast for functions by respondents and their percentage share depending on the situation: healthy athlete (**A**) versus suspected arrhythmia (**B**).

Table S1.2.1 A. Coaches—Healthy athlete

	F 1		F 2		F 3		F 4		F 5		F 6		F 7		F 8		F 9		F 10		F 11	
	VN	%	VN	%	VN	%	VN	%	VN	%	VN	%	VN	%	VN	%	VN	%	VN	%	VN	%
1 P	9	90%	0	0%	1	10%	0	0%	0	0%	0	0%	0	0%	0	0%	0	0%	0	0%	0	0%
2 P	1	10%	8	80%	0	0%	0	0%	0	0%	0	0%	0	0%	1	10%	0	0%	0	0%	0	0%
3 P	0	0%	2	20%	7	70%	0	0%	0	0%	0	0%	0	0%	1	10%	0	0%	0	0%	0	0%
4 P	0	0%	0	0%	2	20%	0	0%	0	0%	0	0%	0	0%	8	80%	0	0%	0	0%	0	0%
5 P	0	0%	0	0%	0	0%	8	80%	0	0%	1	10%	0	0%	0	0%	0	0%	0	0%	1	10%

6 P	0	0%	0	0%	0	0%	1	10%	0	0%	1	10%	0	0%	0	0%	1	10%	0	0%	7	70%
7 P	0	0%	0	0%	0	0%	1	10%	0	0%	6	60%	2	20%	0	0%	0	0%	0	0%	1	10%
8 P	0	0%	0	0%	0	0%	0	0%	0	0%	2	20%	6	60%	0	0%	2	20%	0	0%	0	0%
9 P	0	0%	0	0%	0	0%	0	0%	1	10%	0	0%	1	10%	0	0%	6	60%	1	10%	1	10%
10 P	0	0%	0	0%	0	0%	0	0%	6	60%	0	0%	1	10%	0	0%	1	10%	2	20%	0	0%
11 P	0	0%	0	0%	0	0%	0	0%	3	30%	0	0%	0	0%	0	0%	0	0%	7	70%	0	0%

F, Function; VN, number of votes; P, place.

Table S1.2.1 B. Coaches—suspected arrhythmia

	F 1		F 2		F 3		F 4		F 5		F 6		F 7		F 8		F 9		F 10		F 11	
	VN	%	VN	%	VN	%	VN	%	VN	%	VN	%	VN	%	VN	%	VN	%	VN	%	VN	%
1 P	0	0%	7	70%	1	10%	0	0%	0	0%	1	10%	1	10%	0	0%	0	0%	0	0%	0	0%
2 P	0	0%	1	10%	7	70%	0	0%	0	0%	2	20%	0	0%	0	0%	0	0%	0	0%	0	0%
3 P	1	10%	1	10%	0	0%	0	0%	0	0%	0	0%	8	80%	0	0%	0	0%	0	0%	0	0%
4 P	2	20%	1	10%	1	10%	0	0%	0	0%	6	60%	0	0%	0	0%	0	0%	0	0%	0	0%
5 P	7	70%	0	0%	1	10%	0	0%	0	0%	1	10%	1	10%	0	0%	0	0%	0	0%	0	0%
6 P	0	0%	0	0%	0	0%	1	10%	0	0%	0	0%	0	0%	7	70%	1	10%	0	0%	1	10%
7 P	0	0%	0	0%	0	0%	1	10%	1	10%	0	0%	0	0%	1	10%	1	10%	0	0%	6	60%
8 P	0	0%	0	0%	0	0%	1	10%	1	10%	0	0%	0	0%	0	0%	0	0%	8	80%	0	0%
9 P	0	0%	0	0%	0	0%	6	60%	1	10%	0	0%	0	0%	0	0%	2	20%	1	10%	0	0%
10 P	0	0%	0	0%	0	0%	0	0%	1	10%	0	0%	0	0%	1	10%	6	60%	0	0%	2	20%
11 P	0	0%	0	0%	0	0%	1	10%	6	60%	0	0%	0	0%	1	10%	0	0%	1	10%	1	10%

F, Function; VN, number of votes; P, place.

Table S1.2.2 A. Doctors—healthy athlete

	F 1		F 2		F 3		F 4		F 5		F 6		F 7		F 8		F 9		F 10		F 11	
	VN	%	VN	%	VN	%	VN	%	VN	%	VN	%	VN	%	VN	%	VN	%	VN	%	VN	%
1 P	0	0%	0	0%	0	0%	0	0%	0	0%	1	10%	9	90%	0	0%	0	0%	0	0%	0	0%
2 P	0	0%	0	0%	0	0%	0	0%	0	0%	8	80%	1	10%	0	0%	0	0%	0	0%	1	10%
3 P	0	0%	0	0%	0	0%	0	0%	0	0%	1	10%	0	0%	0	0%	0	0%	2	20%	7	70%
4 P	0	0%	0	0%	1	10%	0	0%	0	0%	0	0%	0	0%	1	10%	0	0%	6	60%	2	20%
5 P	0	0%	0	0%	1	10%	0	0%	0	0%	0	0%	0	0%	8	80%	0	0%	1	10%	0	0%
6 P	0	0%	0	0%	7	70%	1	10%	0	0%	0	0%	0	0%	1	10%	0	0%	1	10%	0	0%
7 P	2	20%	0	0%	1	10%	7	70%	0	0%	0	0%	0	0%	0	0%	0	0%	0	0%	0	0%
8 P	7	70%	2	20%	0	0%	1	10%	0	0%	0	0%	0	0%	0	0%	0	0%	0	0%	0	0%
9 P	1	10%	7	70%	0	0%	1	10%	1	10%	0	0%	0	0%	0	0%	0	0%	0	0%	0	0%
10 P	0	0%	1	10%	0	0%	0	0%	8	80%	0	0%	0	0%	0	0%	1	10%	0	0%	0	0%
11 P	0	0%	0	0%	0	0%	0	0%	1	10%	0	0%	0	0%	0	0%	9	90%	0	0%	0	0%

F, Function; VN, number of votes; P, place.

Table S1.2.2 B. Doctors—suspected arrhythmia

	F 1		F 2		F 3		F 4		F 5		F 6		F 7		F 8		F 9		F 10		F 11	
	VN	%	VN	%	VN	%	VN	%	VN	%	VN	%	VN	%	VN	%	VN	%	VN	%	VN	%

1 P	0	0%	0	0%	0	0%	0	0%	0	0%	0	0%	10	100%	0	0%	0	0%	0	0%	0	0%
2 P	0	0%	0	0%	0	0%	0	0%	0	0%	10	100%	0	0%	0	0%	0	0%	0	0%	0	0%
3 P	0	0%	0	0%	0	0%	0	0%	0	0%	0	0%	0	0%	0	0%	0	0%	1	10%	9	90%
4 P	0	0%	0	0%	0	0%	0	0%	0	0%	0	0%	0	0%	0	0%	0	0%	9	90%	1	10%
5 P	0	0%	2	20%	8	80%	0	0%	0	0%	0	0%	0	0%	0	0%	0	0%	0	0%	0	0%
6 P	1	10%	7	70%	2	20%	0	0%	0	0%	0	0%	0	0%	0	0%	0	0%	0	0%	0	0%
7 P	7	70%	1	10%	0	0%	1	10%	0	0%	0	0%	0	0%	1	10%	0	0%	0	0%	0	0%
8 P	1	10%	0	0%	0	0%	8	80%	0	0%	0	0%	0	0%	1	10%	0	0%	0	0%	0	0%
9 P	1	10%	0	0%	0	0%	1	10%	0	0%	0	0%	0	0%	8	80%	0	0%	0	0%	0	0%
10 P	0	0%	0	0%	0	0%	0	0%	1	10%	0	0%	0	0%	0	0%	9	90%	0	0%	0	0%
11 P	0	0%	0	0%	0	0%	0	0%	9	90%	0	0%	0	0%	0	0%	1	10%	0	0%	0	0%

F, Function; VN, number of votes; P, place.

Table S1.2.3 A. Athletes—healthy athlete

	F 1		F 2		F 3		F 4		F 5		F 6		F 7		F 8		F 9		F 10		F 11	
	VN	%	VN	%	VN	%	VN	%	VN	%	VN	%	VN	%	VN	%	VN	%	VN	%	VN	%
1 P	95	95%	5	5%	0	0%	0	0%	0	0%	0	0%	0	0%	0	0%	0	0%	0	0%	0	0%
2 P	5	5%	90	90%	5	5%	0	0%	0	0%	0	0%	0	0%	0	0%	0	0%	0	0%	0	0%
3 P	0	0%	4	4%	86	86%	4	4%	0	0%	0	0%	0	0%	5	5%	0	0%	0	0%	0	0%
4 P	0	0%	1	1%	5	5%	3	3%	0	0%	0	0%	0	0%	91	91%	0	0%	0	0%	0	0%
5 P	0	0%	0	0%	4	4%	85	85%	1	1%	0	0%	0	0%	3	3%	0	0%	0	0%	6	6%
6 P	0	0%	0	0%	0	0%	4	4%	10	10%	2	2%	0	0%	1	1%	0	0%	0	0%	85	85%
7 P	0	0%	0	0%	0	0%	4	4%	89	89%	2	2%	0	0%	0	0%	0	0%	0	0%	5	5%
8 P	0	0%	0	0%	0	0%	0	0%	0	0%	78	78%	13	13%	0	0%	3	3%	2	2%	4	4%
9 P	0	0%	0	0%	0	0%	0	0%	0	0%	11	11%	67	67%	0	0%	17	17%	5	5%	0	0%
10 P	0	0%	0	0%	0	0%	0	0%	0	0%	5	5%	17	17%	0	0%	65	65%	13	13%	0	0%
11 P	0	0%	0	0%	0	0%	0	0%	0	0%	2	2%	3	3%	0	0%	15	15%	80	80%	0	0%

F, Function; VN, number of votes; P, place.

Table S1.2.3 B. Athletes—Suspicion of arrhythmia

	F 1		F 2		F 3		F 4		F 5		F 6		F 7		F 8		F 9		F 10		F 11	
	VN	%	VN	%	VN	%	VN	%	VN	%	VN	%	VN	%	VN	%	VN	%	VN	%	VN	%
1 P	76	76%	24	24%	0	0%	0	0%	0	0%	0	0%	0	0%	0	0%	0	0%	0	0%	0	0%
2 P	15	15%	76	76%	9	9%	0	0%	0	0%	0	0%	0	0%	0	0%	0	0%	0	0%	0	0%
3 P	8	8%	0	0%	79	79%	5	5%	0	0%	8	8%	0	0%	0	0%	0	0%	0	0%	0	0%
4 P	0	0%	0	0%	0	0%	3	3%	0	0%	2	2%	90	90%	0	0%	0	0%	0	0%	5	5%
5 P	1	1%	0	0%	5	5%	3	3%	0	0%	87	87%	4	4%	0	0%	0	0%	0	0%	0	0%

6 P	0	0%	0	0%	0	0%	1	1%	0	0%	3	3%	6	6%	0	0%	0	0%	0	0%	90	90%
7 P	0	0%	0	0%	6	6%	86	86%	0	0%	0	0%	0	0%	0	0%	0	0%	2	2%	5	5%
8 P	0	0%	0	0%	1	1%	2	2%	1	1%	0	0%	0	0%	6	6%	21	21%	70	70%	0	0%
9 P	0	0%	0	0%	0	0%	0	0%	8	8%	0	0%	0	0%	86	86%	0	0%	6	6%	0	0%
10 P	0	0%	0	0%	0	0%	0	0%	75	75%	0	0%	0	0%	8	8%	4	4%	13	13%	0	0%
11 P	0	0%	0	0%	0	0%	0	0%	16	16%	0	0%	0	0%	0	0%	75	75%	9	9%	0	0%

F, Function; VN, number of votes; P, place.

Table S1.3. Reasons for preferential use of wrist-worn optical heart rate monitors (OHRMs) versus chest strap HRMs (SHRMs) by athletes, coaches, and doctors, assuming that both types of HRM have the same functions and the same resistance to artifacts.

Respondent	Sport discipline	Gender [Male/ Female]	Age [years]	Experience with OHRMs [years]	Experience with SHRMs [years]	R1	R2	R3	R4	R5	R6	R7
T 1	N/A	M	45	0.5	6.5	1	1	1	1	1	2	1
T 2	N/A	M	51	3.5	6	1	1	1	1	1	1	2
T 3	N/A	M	48	2	5	1	1	2	1	1	2	2
T 4	N/A	M	54	3.5	5.5	2	1	1	1	1	2	2
T 5	N/A	M	26	1	4	1	1	2	1	1	2	2
T 6	N/A	M	51	2.5	8	1	1	1	1	2	2	2
T 7	N/A	M	46	3	8.5	1	2	1	1	2	2	2
T 8	N/A	M	36	3	9.5	1	1	2	1	2	2	2
T 9	N/A	M	60	2	10	1	1	1	1	1	2	2
T 10	N/A	M	34	4	3	2	1	2	1	2	2	2
				OHRMs		80%	90%	60%	100%	60%	10%	10%
				SHRMs		20%	10%	40%	0%	40%	90%	90%
D 1	N/A	M	43	0.5	4	1	1	2	1	1	2	2
D 2	N/A	M	51	4	5	1	1	1	1	2	2	2
D 3	N/A	M	39	1.5	5.5	2	1	1	1	1	2	2
D 4	N/A	M	59	2	7	1	1	1	1	1	1	2
D 5	N/A	M	49	1.5	6.5	1	1	2	1	1	2	2
D 6	N/A	M	60	4	9	1	1	1	1	2	2	2
D 7	N/A	M	55	3.5	5.5	1	1	1	1	2	2	2
D 8	N/A	M	33	1	5	1	1	2	1	1	2	2
D 9	N/A	M	57	3	4.5	1	1	1	1	2	2	2
D 10	N/A	M	53	3	10	2	1	1	1	1	2	1
				OHRMs		80%	100%	70%	100%	60%	10%	10%
				SHRMs		20%	0%	30%	0%	40%	90%	90%
A	R 1	M	35	0.5	5	1	1	1	1	1	2	2
A	R 2	M	31	3	2	1	1	1	2	2	2	2
A	R 3	F	25	2	2.5	1	1	1	1	1	2	2
A	R 4	M	35	0.75	6	1	1	1	1	1	2	2
A	R 5	F	37	4	5	1	2	1	1	1	2	2
A	R 6	F	39	1	5.75	2	1	1	1	2	2	2

A	R 7	M	36	2	4	2	1	2	1	1	1	2
A	R 8	M	41	1	9.5	1	1	1	1	1	2	2
A	R 9	M	43	3	8	1	1	2	1	1	2	2
A	R 10	F	28	3	2.5	1	1	1	1	1	2	2
A	R 11	M	35	2	9	1	1	1	1	2	1	2
A	R 12	M	29	1	3.5	1	1	1	1	1	2	2
A	R 13	M	36	1.25	3.25	1	1	1	1	1	2	2
A	R 14	M	37	1	10	1	1	1	1	1	2	2
A	R 15	F	42	1.25	5.25	1	1	1	1	1	2	2
A	R 16	M	36	3	8	1	1	1	1	2	2	2
A	R 17	M	29	1	4.5	2	1	1	1	1	2	2
A	R 18	M	29	2	5.5	1	1	1	1	1	1	2
A	R 19	F	39	1	5	2	1	1	1	2	2	2
A	R 20	M	27	3	2.75	1	1	1	1	1	2	2
A	R 21	M	34	2	3	1	1	1	1	1	2	2
A	R 22	F	39	2	8	2	1	1	1	1	2	2
A	R 23	M	41	0.75	7.5	1	1	2	1	1	2	2
A	R 24	M	21	0.25	2	1	1	2	1	1	2	2
A	R 25	M	43	1	5.25	1	1	2	1	1	2	2
A	R 26	F	44	3	6.5	2	1	1	1	2	1	2
A	R 27	M	28	1	3.25	1	1	1	2	1	2	2
A	R 28	M	31	0.5	3	1	1	1	1	2	2	2
A	R 29	M	32	1.25	4	1	1	1	2	1	2	2
A	R 30	M	35	1	7	1	1	1	1	1	2	2
A	R 31	F	38	0.25	6.5	2	1	2	1	2	2	2
A	R 32	M	36	1	4.25	1	2	1	1	2	1	2
A	R 33	M	38	1.5	9	1	1	1	1	2	2	2
A	R 34	M	28	1	3.75	1	1	1	1	1	2	2
A	R 35	F	31	1	6.5	1	1	1	1	1	2	2
A	R 36	M	27	3.5	2.5	1	1	2	1	2	2	2
A	R 37	F	34	1	7.5	1	1	2	1	1	2	2
A	R 38	M	34	1	8.5	1	1	1	1	1	2	1
A	R 39	M	40	2	4.25	1	1	2	1	2	1	2
A	R 40	F	42	2	6.25	1	1	2	1	1	2	2
A	R 41	M	57	1	10	1	1	1	1	1	2	2
A	R 42	F	26	3	2	1	1	1	1	1	2	2
A	R 43	F	33	2	5.5	1	1	1	1	1	2	2
A	R 44	M	30	3	2.5	1	1	1	1	1	2	2
A	R 45	F	33	1	3.5	1	1	1	1	1	2	2
A	R 46	F	36	2	5	2	1	1	1	2	2	2
A	R 47	M	44	1	5.25	1	1	1	1	1	2	2
A	R 48	M	33	2.5	6.5	1	1	2	2	2	2	2
A	R 49	M	38	2	9.25	1	1	1	1	2	2	2
A	R 50	F	36	1	7.5	1	1	1	2	1	2	2
A	R 51	M	36	3	7	2	1	1	1	1	2	2

A	R 52	M	50	0.75	10	1	1	1	1	2	2	2
A	R 53	F	45	0.5	6	1	1	1	1	2	2	2
A	R 54	M	37	2	5	1	1	1	1	1	2	1
A	R 55	M	37	2	6	1	1	1	1	2	2	2
A	R 56	M	38	0.5	4.25	1	1	1	1	1	2	2
A	R 57	M	40	0.25	8	1	1	2	1	1	2	1
A	R 58	M	35	0.25	4	1	1	1	1	1	2	2
A	R 59	F	36	2.5	6	1	2	1	1	1	2	2
A	R 60	F	25	1.75	3.25	1	1	1	2	1	2	2
A	R 61	M	31	0.75	5.5	1	1	1	1	1	2	2
A	R 62	M	25	1.5	5.25	1	1	1	1	1	2	2
A	R 63	F	31	0.25	3.5	1	1	1	1	1	2	1
A	R 64	M	35	2	8	1	1	1	1	1	2	2
A	R 65	M	28	2	2.25	1	1	1	1	2	2	2
A	R 66	M	29	3	2.5	2	1	2	1	2	2	2
A	R 67	M	38	3	6	1	1	2	1	2	2	2
A	R 68	M	39	1	9	1	1	2	1	2	1	2
A	R 69	F	30	1	4.25	1	1	1	1	1	2	2
A	R 70	M	51	1	9	1	1	1	1	2	2	2
A	R 71	F	22	0.5	3	1	1	1	1	1	2	2
A	R 72	M	34	2	2	1	1	1	1	1	2	2
A	R 73	F	34	1	9.5	1	2	2	1	2	2	2
A	R 74	F	26	2.5	4.5	2	1	1	1	2	2	2
A	R 75	M	43	2	9.5	1	1	1	1	1	1	2
A	R 76	F	24	1	2.25	1	1	1	1	2	2	2
A	C 1	M	24	1.5	2.25	1	1	1	1	1	2	2
A	C 2	M	37	3	7	1	1	2	1	2	2	2
A	C 3	M	27	2	5	1	1	1	1	1	2	2
A	C 4	M	48	0.5	8.5	2	1	1	1	1	2	2
A	C 5	M	26	3	3	1	1	1	1	2	2	2
A	C 6	M	37	1	9	1	1	2	1	1	2	2
A	C 7	M	39	3	10	1	1	1	1	1	2	2
A	C 8	F	55	1	6.25	1	1	2	1	1	2	2
A	C 9	M	38	0.75	7	1	1	2	2	1	2	2
A	C 10	F	38	1	7.25	1	1	1	1	1	2	2
A	C 11	M	40	2	5.25	1	1	1	1	1	2	2
A	C 12	M	30	2	3.25	1	1	1	1	1	2	2
A	C 13	M	30	2	3.25	1	1	2	1	2	2	2
A	C 14	M	33	2	4.5	1	1	1	1	2	2	2
A	TriA 1	M	40	4	7	1	1	2	1	2	2	2
A	TriA 2	F	24	0.75	2.25	1	1	1	1	2	2	2
A	TriA 3	M	27	1	4	1	1	1	1	1	1	2
A	TriA 4	M	32	1.75	2.5	1	1	1	1	1	2	2
A	TriA 5	M	39	1.75	6.75	1	1	1	1	1	2	2
A	TriA 6	M	32	1.25	4.25	1	2	1	1	1	2	2

A	TriA 7	M	39	2	9	1	1	2	1	1	1	2
A	TriA 8	M	40	1	10	1	1	1	1	1	2	2
A	TriA 9	M	41	0.5	8.5	1	1	2	1	1	2	2
A	TriA 10	M	38	1	6.25	1	1	2	1	2	1	2
OHRMs						88%	95%	75%	93%	67%	11%	4%
SHRMs						12%	5%	25%	7%	33%	89%	96%

N/A, not applicable; R, reason; R1, Comfort of use during training; R2, Comfort of use around the clock; R3, Battery life; R4, Skin abrasions from the strap belt; R5, Trend/Fashion; R6, Habit; R7, Confidence in the accuracy of indications. OHRM, optical HRM; SHRM, strap HRM; T, trainer (coach); A, athlete; triA, triathlete; C, cyclist; D, doctor; R, runner.
